# Tibial plateau fracture and RNA sequencing with osteogenesis imperfecta: a case report

**DOI:** 10.3389/fendo.2023.1164386

**Published:** 2023-05-09

**Authors:** Yixiao Chen, Guoqing Li, Liangchen Wei, Jian Weng, Su Liu, Mingxi Gu, Pei Liu, Yuanchao Zhu, Ao Xiong, Hui Zeng, Fei Yu

**Affiliations:** ^1^ Department of Bone and Joint Surgery, Peking University Shenzhen Hospital, Shenzhen, China; ^2^ National and Local Joint Engineering Research Center of Orthopaedic Biomaterials, Peking University Shenzhen Hospital, Shenzhen, China

**Keywords:** osteogenesis imperfecta, RNA transcriptome sequencing, tibial plateau fracture, diagnosis, surgery

## Abstract

Osteogenesis imperfecta (OI) is a hereditary skeletal dysplasia with an incidence of approximately 1:15,000 to 20,000. OI is usually caused by the mutation of COL1A1 and COL1A2, which would encode the α-chain of type I collagen. OI is clinically characterized by decreased bone mass, increased risk of bone fragility, blue sclerae, and dentinogenesis.

**Case presentation:**

A 29-year-old male patient was diagnosed with right tibial plateau fracture caused by slight violence. Physical examination revealed the following: height, 140 cm; weight, 70 kg; body mass index (BMI), 35.71 kg/m^2^; blue sclera and barrel chest were observed. X-ray examination showed left convex deformity of the thoracic vertebrae with reduced thoracic volume. Laboratory examinations revealed a decrease in both vitamin D and blood calcium levels. Bone mineral density (BMD) was lower than the normal range. After the preoperative preparation was completed, the open reduction and internal fixation of the right tibial plateau fracture were performed. Meanwhile, whole blood samples of this OI patient and the normal control were collected for RNA transcriptome sequencing. The RNA sequence analysis revealed that there were 513 differentially expressed genes (DEGs) between this OI patient and the normal control. KEGG-enriched signaling pathways were significantly enriched in extracellular matrix (ECM)–receptor interactions.

**Conclusion:**

In this case, DEGs between this OI patient and the normal control were identified by RNA transcriptome sequencing. Moreover, the possible pathogenesis of OI was also explored, which may provide new evidence for the treatment of OI.

## Introduction

Osteogenesis imperfecta (OI) is an inherited skeletal dysplasia disease, which is characterized by low bone mass, increased bone fragility, and dentinogenesis imperfecta. Moreover, OI patients may have an array of extra-skeletal features, including blue sclera, hearing loss, and pulmonary function impairment (1). OI is an autosomal dominant genetic disease generally caused by mutations of COL1A1 and COL1A2, which could encode the chain of type I collagen (2). The mutations are usually divided into four types from mild to severe according to clinical manifestations, namely, type I: mild non-deforming phenotype; type II: the most severe and usually resulting in perinatal death; type III: severe non-lethal progressive deforming phenotype; and type IV: intermediate between types I and III (3, 4). The type I collagen could pre-mineralize bone matrixes, while the abnormal synthesis of type I collagen would result in the loss of bone mass and increased fracture susceptibility (5). However, the specific molecular mechanism of OI remains unclear, the treatment methods of which need to be improved. In clinical settings, OI patients with fractures can be treated by surgical or conservative methods, with the former playing an important role in the treatment of most fractures. Currently, the treatment for OI mainly focuses on improving bone strength and preventing bone loss, while the drugs used to treat OI usually include bisphosphonates, denosumab, teriparatide, and vitamin D (6).

At present, whole blood RNA transcriptome sequencing has become a major approach to discover differently expressed genes (DEGs), pathways, biomarkers, and therapies with high sensitivity and robustness (7). In this case report, a 29-year-old male patient was diagnosed with OI with a right tibial plateau fracture and underwent surgical treatment in our department. Whole blood RNA transcriptome sequencing was used to explore the new pathogenesis.

## Case report

**Figure 1:**
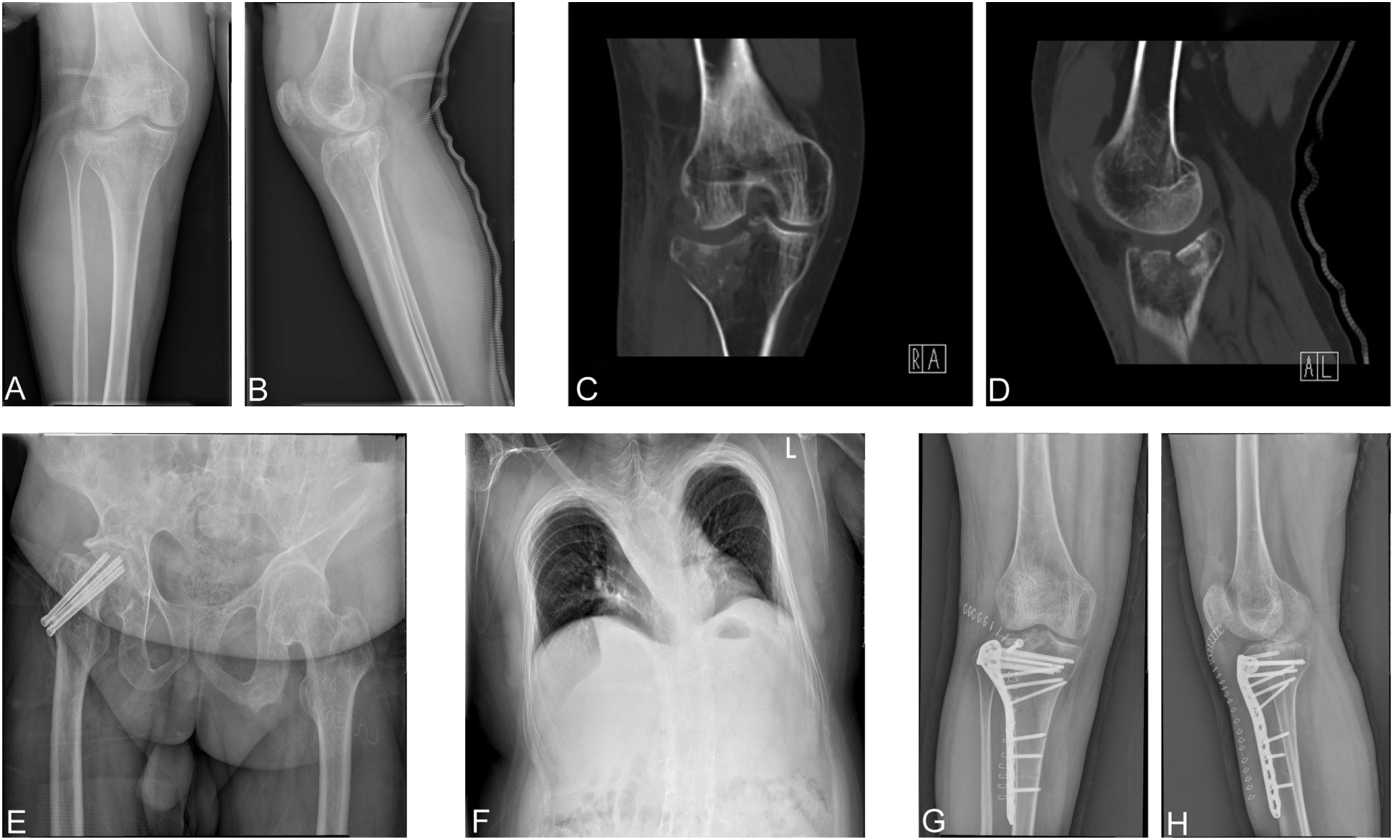
**(A, B)** Preoperative positive and lateral x-rays of the knee joint. **(C, D)** Preoperative CT plain scan of the right knee joint. **(E)** Anteroposition radiographs of the right hip. **(F)** X-ray of the chest. **(G, H)** Postoperative right knee joint anteroposterior and lateral x-ray showed good alignment of the broken end and internal fixation in place.

A 29-year-old man was admitted to our department; he primarily complained of pain, swelling, and limited mobility of the right knee joint during his bath. After the imaging examinations of the right knee joint, including x-ray (**Figures 1A, B**) and CT (**Figures 1C, D**), the patient was diagnosed with right tibial plateau fractures (Schatzker type II).

The patient has suffered from three fractures in both upper and lower limbs, namely, a left humeral condyle fracture at 8 years old, a right ulna olecranon fracture at 16 years old, and a right femoral neck fracture at 21 years old. These fractures were treated with surgery including left humeral condyle and right ulna olecranon open reduction with internal fixation and right femoral neck fracture closed reduction and internal fixation (**Figure 1E**), respectively. The patient recovered from such fractures postoperatively. Moreover, he did not receive any anti-bone catabolic medication, such as calcium, vitamin D, or bisphosphonates, previously. In addition, he does not have any disease, nor does he take any drugs that might cause osteoporosis and fracture apart from OI. Meanwhile, the patient’s father and mother had not been diagnosed with OI and had no previous history of fractures or osteoporosis.

Physical examination revealed the following from the patient: height, 140 cm; body weight, 70 kg; and body mass index, 35.71 kg/m^2^. Furthermore, blue sclera and a barrel chest were observed and x-ray images showed left convex deformity of the thoracic vertebrae with reduced thoracic volume (**Figure 1F**). In addition, there was no evidence of hearing impairment, dysplasia of teeth, cardiac murmurs, or respiratory difficulty, and there are no abnormalities in neurological examination. Results of laboratory examinations, including routine blood examination, blood coagulation function, kidney function, liver function, and blood gas analysis, were within normal ranges. Cardiac color ultrasound showed no obvious abnormalities, but the serum level of the patient including both 25-hydroxyvitamin D (**Table 1**) and calcium ions (**Table 2**) was decreased. The BMD of the OI patient was measured by dual-energy x-ray absorptiometry, and the results showed that the *Z* value was −1.8, which indicated that the BMD was lower than the normal range. The radiographic features and clinical symptoms were consistent with the characteristics of OI as well. Therefore, the patient was diagnosed with OI type I.

**Table 1:** Osteoporosis’ four parameters.

Parameters	Result	Normal range
β-CTX	0.28 ng/mL	0.04-0.78 ng/mL
P1NP	20.35 ng/mL	9.1-76.2 ng/mL
Vit D	17.13 ng/mL	>20 ng/mL
Osteocalcin	23.40 ng/mL	6.0-24.7 ng/mL

β-CTX, β-C-terminal telopeptide of type I collagen; TPINP, total N-terminal propeptide of type I procollagen; Vit D, 25-hydroxyvitamin D.

**Table 2:** Electrolyte’s six parameters.

Parameters	Result	Normal range
K	3.76 mmol/L	3.5-5.3 mmol/L
CL	101.7 mmol/L	99-100 mmol/L
Mg	0.9 mmol/L	0.75-1.02 mmol/L
Na	139 mmol/L	137-147 mmol/L
Ca	2.09 mmol/L	2.11-2.52 mmol/L
P	1.08 mmol/L	0.85-1.51 mmol/L

After preoperative preparation was completed, the patient underwent open reduction and internal fixation for the right tibial plateau fracture. During surgery, a compression fracture and collapse of the right lateral tibial plateau were seen in the patient. After the restoration of the lateral tibia plateau, the allograft bone was filled, and the internal fixation was performed with anatomic bone plates (**Figures 1G, H**). After operation, the patient was safely returned to the ward. When the patient became stable postoperatively, whole blood was collected for RNA sequencing with permission and written informed consent from the OI patient. In addition, a 26-year-old man was selected as a normal control and signed informed consent. The normal control was 177 cm tall, weighed 70 kg, had a normal development, was in good physical condition, and had a BMD *Z* value of −0.9, which was in the normal range. Therefore, he can be used as a control.

The 2-ml whole blood samples of the OI patient and the normal control were respectively collected following the addition of 6 ml of Trizol and were submitted to Novogene (Beijing, https://en.novogene.com/) for library construction. After meeting library qualifications, sample sequencing was conducted using Illumina Novaseq 6000. Finally, all results were applied to subsequent bioinformatics analysis. The Gene Ontology (GO) enrichment analysis of DEGs was implemented by the clusterProfiler R package (3.8.1), during which the gene length bias was corrected. GO terms with a corrected *p*-value of less than 0.05 were considered significantly enriched by DEGs, and statistical enrichment of DEGs in the KEGG pathway was also analyzed using the clusterProfiler R package (3.8.1).

**Figure 2:**
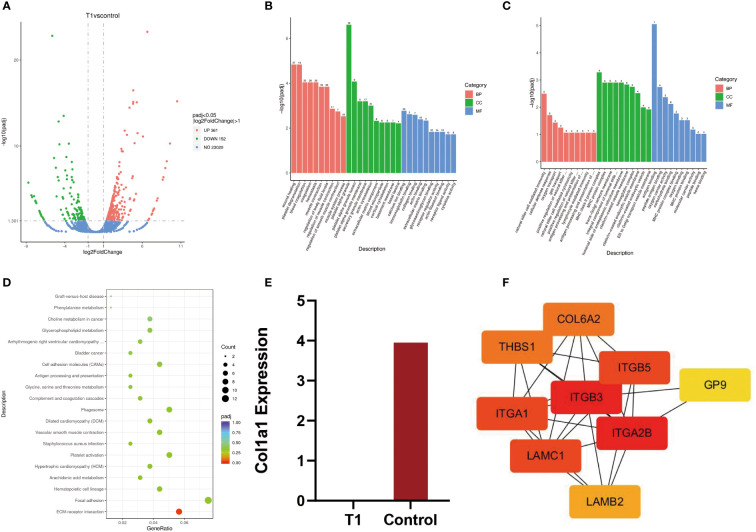
**(A)** Volcanic map of differential genes between patients and normal people. **(B, C)** Upregulated genes and downregulated genes were analyzed by GO enrichment between patients and normal controls. **(D)** The KEGG pathway between patients and normal controls. **(E)** T1 = patient, control = normal control, quantitative analysis of transcripts, **(F)** Protein–protein interaction network.

The results of whole blood transcriptome sequencing showed that there were 513 DEGs between the OI patient and the normal control (**Figure 2A**). Among them, 361 genes were upregulated, while 152 genes were downregulated. The top five most significantly upregulated genes were *RAP1GAP, TMEM176B, BTNL3, TMEM176A*, and *IGKV2-30* (*p*-value 2.28E-28, 4.29E-21, 1.09E-19, 1.59E-19, and 2.12E-19, respectively). The top five significantly downregulated genes were *RPL9P9, RPS28P7, HLA-DQA1, HLA-DRB1*, and *DDX11L10* (*p*-value 1.34E-27, 1.20E-17, 6.06E-17, 3.87E-14, and 4.87E-14, respectively).

GO analysis is composed of biological process (BP), cellular component (CC), and molecular function (MF). The enrichment results of 30 upregulated genes by GO analysis showed that wound healing was mainly enriched in BP, platelet α particles in CC, and calcium ion binding in MF. In addition, the GO enrichment results of 30 downregulated genes showed that natural killer cell-mediated immunity was mainly enriched in BP, MHC protein complexes in CC, and antigen-binding in MF (**Figures 2B, C**).

KEGG is a common database for gene research, and it could be used to identify significantly enriched signaling pathways (8). In this study, the results of KEGG signaling pathways were mainly enriched in ECM–receptor interaction (**Figure 2D**). The genes enriched in this pathway included *ITGA2B, LAMC1, LAMB2, COL6A2, ITGB3, THBS1, ITGB5, GP9*, and *ITGA1*. Among them, eight genes were upregulated and one gene was downregulated. The expression data of these genes are listed in **Table 3**.

**Table 3:** ECM–receptor interaction enrichment genes.

Gene name	T1	Control	Pvalue	Gene_description
*ITGA2B*	14200.5	1434.29	1.74E-13	Integrin subunit alpha 2b
*LAMC1*	258.14	27.47	1.49E-10	Iaminin subunit gamma
*LAMB2*	73.25	5.82	2.95E-08	Laminin subunit gamma 1
*ITGB3*	1841.71	434.77	1.49E-06	Integrin subunit beta 3
*THBS1*	1661.62	411.45	3.10E-06	Thrombospondin 1
*ITGB5*	1341.06	367.3	1.44E-05	Integrin subunit beta 5
*GP9*	995.3	339.82	0.0003	Glycoprotein IX platelet
*ITGA1*	102.07	29.97	0.0005	Integrin subunit alpha 1
*COL6A2*	176.5	850.41	3.59E-07	Collagen type VI alpha 2 chain

The results of DEGs showed that there was no expression of COL1A1 in the patient. However, the expression of COL1A1 in the normal control was upregulated, with fourfold expression (*p*-value 0.26 > 0.05) (**Figure 2E**).

The protein–protein interaction (PPI) network was plotted based on the STRING database and inserted into the Cytoscape software (v 3.9.1) for visualization. Cytohub plug-in was used to analyze the ECM–receptor interaction-related genes, including *ITGA2B, LAMC1, LAMB2, COL6A2, ITGB3, THBS1, ITGB5, GP9*, and *ITGA1* (**Figure 2F**). According to the correlation degree, these genes can be ranked as *ITGB3, ITGA2B, ITGA1, LAMC1, COL6A2, THBS1, LAMB2*, and *GP9*. The hub genes with high values were *ITGB3* and *ITGA2B*.

## Discussion

OI is an inherited disease caused by abnormal changes in type I collagen, with an incidence of 1:15,000 to 20,000 (9). These abnormal changes result in the excessive modification of collagen molecules and thinner collagen fibers. In OI patients, the increased risk of fractures resulted from the decreased trabecular number, connections, trabecular thickness, bone volume, cortical thickness, and mechanical anisotropy and increased porosity (10). In addition, the deficiency of serum 25-hydroxyvitamin D is a recognized risk factor for osteoporosis and fractures (11). For this patient, the vitamin D deficiency might have contributed to bone mass loss and multiple fractures.

The severity of symptoms varies significantly among different OI patients. Patients with mild symptoms have normal quality of life with minor fractures, while those with severe symptoms may be accompanied by severe fractures, physical disability, or even a reduced life span (12). OI might also cause coagulation dysfunction, airway obstruction, delayed wound healing, and other conditions (13). Most OI patients are more likely to suffer from multiple fractures in their childhood. Just like this OI patient, multiple fractures but no life-threatening symptoms occurred previously. In order to avoid stress fractures at the edges of the plates or screws, surgeons prefer to use intramedullary nail fixation rather than plate or screw fixation (14). As for the intra-articular fractures in this OI patient, surgical treatment was selected to restore the normal joint structure and reestablish a smooth joint surface, in an attempt to avoid such long-term complications as traumatic arthritis. After joint surface uniformity was achieved and allograft bone was applied, the plate and screw for rigid fixation were placed intraoperatively. In addition, the patient did not have any complications, such as recurrence of fractures and deep venous thrombosis of the lower limbs, during the treatment in our department.

GO and KEGG were applied for enrichment analysis. The enrichment results of upregulated genes by GO analysis showed that wound healing was the main process in BP, platelet α particles in CC, and calcium ion binding in MF. Moreover, GO enrichment results of downregulated genes showed that natural killer cell-mediated immunity was the main factor in BP, MHC protein complex in CC, and antigen-binding in BP. Among these KEGG enriched pathways, ECM–receptor interaction was the main enriched pathway, and nine genes (*ITGA2b, LAMC1, LAMB2, COL6A2, ITGB3, THBS1, ITGB5, GP9*, and *ITGA1*) were enriched.

OI is a common systemic connective tissue disorder caused by abnormal synthesis and variants in exon encoding and synthesis of type I collagen, which is essential for maintaining normal ECM functions (15, 16). In this case, transcript quantification revealed that compared with the normal control, there was no expression of CoL1A1 in this OI patient, which might be the pathogenic factor in the patient. ECM refers to a category of macromolecules composed of collagen, glycoproteins, proteoglycans, and hyaluronic acid (17). It can also participate in various functions and regulate the maturation of bones. The bone matrix consists of organic compounds (40%) and inorganic compounds (60%). The organic ECM is composed of type I collagen (90%) and non-collagen, while type I collagen can participate in the variable metabolisms of the bones (7, 18). However, the alterations of type I collagen would result in a decrease in the quality of ECM and increased the fragility of bones (19). Mutations of type I collagen induce abnormal protein secretion into the matrix that interferes with fibrillogenesis, collagen matrix or collagen cell interaction, and mineralization. Hence, structural mutations in the ECM are more severe than the deficiency of the matrix (20). In addition, the alterations in ECM components could block ECM osteocyte signaling transduction, which leads to the disruption of BMD or bone microarchitecture (21).

ECM functions require activations of ECM receptors, and the integrin family is the main recognition molecule (22). Integrins are composed of 18α and 8β subunits, which constitute heterodimeric transmembrane receptors that can assemble noncovalently into 24 heterodimers (23). The deficiency in α2 and α11 subunits in hMSC would reduce the adhesion, diffusion, and migration of type I collagen. It also affects integrin-mediated signal transduction (24). In addition, the abnormal expression of α2 integrin and α2β1 heterodimer could be observed in patients with osteoporosis (21). In this study, the hub genes *ITGB3* and *ITGA2B* were identified through a PPI network, and the changes associated with these genes may be involved in the pathogenesis of OI.

Nevertheless, there are still some potential limitations in this study. Firstly, a case report study might not allow us to draw conclusive statements. However, the significant DEGs detected between the OI patient and the normal control should be highlighted. Secondly, this case report was only generated from a single center, which prevented us from obtaining definitive conclusions. Therefore, it is necessary to conduct further prospective multicentric randomized controlled trials with a larger sample size and long-term follow-up based on a large-scale population, with the aim of fully understanding the benefits of the treatment for patients with OI combined with fractures.

In conclusion, to provide more in-depth insights into the molecular mechanisms and treatment of OI, this case report described an OI patient with right tibia plateau fractures. Moreover, RNA transcriptome sequencing was also performed to analyze DEGs. Furthermore, a long period of follow-up with more details for instruction on rehabilitation should be required for the patient to avoid unnecessary muscle atrophy and dyskinesia.

## Data availability statement

The datasets for this article are not publicly available due to concerns regarding participant/patient anonymity. Requests to access the datasets should be directed to the corresponding author.

## Ethics statement

The studies involving human participants were reviewed and approved by Peking University Shenzhen Hospital ethics committee. The patients/participants provided their written informed consent to participate in this study. Written informed consent was obtained from the individual for the publication of any potentially identifiable images or data included in this article.

## Author contributions

YC, GL, and LW: Investigation, Methodology, Data curation, Formal analysis, Writing-original draft and Writing-review and editing. JW and MG: Methodology. SL: Investigation and Methodology. YZ and PL: Methodology. AX: Investigation. HZ: Investigation, Conceptualization, Supervision, Funding acquisition, Resources, Review, and Editing. FY: Investigation, Resources, and review. All authors contributed to the article and approved the submitted version.
